# Identification of Quorum-Sensing Molecules of *N*-Acyl-Homoserine Lactone in *Gluconacetobacter* Strains by Liquid Chromatography-Tandem Mass Spectrometry

**DOI:** 10.3390/molecules24152694

**Published:** 2019-07-24

**Authors:** Ling-Pu Liu, Long-Hui Huang, Xiao-Tong Ding, Lin Yan, Shi-Ru Jia, Yu-Jie Dai, Yan-Yan Xie, Cheng Zhong

**Affiliations:** 1 State Key Laboratory of Food Nutrition & Safety, Tianjin University of Science & Technology, Tianjin 300457, China; 2 Key Laboratory of Industrial Fermentation Microbiology, (Ministry of Education), Tianjin University of Science & Technology, Tianjin 300457, China

**Keywords:** *N*-acyl-homoserine lactones (AHLs), quorum-sensing system, *Gluconacetobacter* strains

## Abstract

Many Gram-negative bacteria can regulate gene expression in a cell density-dependent manner via quorum-sensing systems using *N*-acyl-homoserine lactones (AHLs), which are typical quorum-sensing signaling molecules, and thus modulate physiological characteristics. *N*-acyl-homoserine lactones are small chemical molecules produced at low concentrations by bacteria and are, therefore, difficult to detect. Here, a biosensor system method and liquid chromatography-tandem mass spectrometry were combined to detect and assay AHL production. As demonstrated by liquid chromatography-tandem mass spectrometry, *Gluconacetobacter xylinus* CGMCC No. 2955, a Gram-negative acetic acid-producing bacterium and a typical bacterial cellulose (BC) biosynthesis strain, produces six different AHLs, including *N*-acetyl-homoserine lactone, *N*-butanoyl-homoserine lactone, *N*-hexanoyl-homoserine lactone, *N*-3-oxo-decanoyl-homoserine lactone, *N*-dodecanoyl-homoserine lactone, and *N*-tetradecanoyl-homoserine lactone. *Gluconacetobacter sp.* strain SX-1, another Gram-negative acetic acid-producing bacterium, which can synthesize BC, produces seven different AHLs including *N*-acetyl-homoserine lactone, *N*-butanoyl-homoserine lactone, *N*-hexanoyl-homoserine lactone, *N*-3-oxo-octanoyl-homoserine lactone, *N*-decanoyl-homoserine lactone, *N*-dodecanoyl-homoserine lactone, and *N*-tetradecanoyl-homoserine lactone. These results lay the foundation for investigating the relationship between BC biosynthesis and quorum-sensing systems.

## 1. Introduction

Bacterial quorum-sensing systems are regulatory mechanisms that perceive cell density and activate cognate gene expression to regulate bacterial group behavior [1]. Bacteria can regulate group behavior by cell-to-cell communication; this inter-bacterial communication system, known as quorum sensing, utilizes hormones, such as *N*-acyl-homoserine lactones (AHLs), oligopeptides, and furanosyl borate diester, to regulate bacterial gene expression and, subsequently, alter physiological characteristics [2,3,4,5]. Bacterial quorum sensing was first reported by Nealson et al. [6] in 1970. They discovered that there was a positive correlation between the cell densities of *Vibrio fischeri* and their bioluminescence. When the cell densities reach a certain threshold, *Vibrio fischeri* can emit fluorescence. In contrast, the fluorescence can disappear with a decreased cell density. *Vibrio fischeri* synthesizes small signal molecules called autoinducers that accumulates in the environment. At low cell densities, few autoinducers are present, and no bioluminescence enzymes accumulate. With an increasing cell density, a threshold concentration of autoinducers is reached, and the bioluminescence enzymes begin to be synthesized [6]. In Gram-negative bacteria, the signal molecules usually employed are AHLs, which affect functions such as swimming and swarming motility, Ti conjugal plasmid transfer, biofilm formation, antibiotic biosynthesis, and virulence [7,8,9,10]. *N*-acyl-homoserine lactones are synthesized by an AHL synthetase that belongs to the LuxI protein family. The *luxR* genes are usually found near to a *luxI* gene, which are coded for the LuxI protein. These LuxR proteins are known to have nine invariant amino acids, including W57, Y61, D70, P71, W85, G113, E178, L182, and G188 [11]. *Gluconacetobacter* strains have been identified as bacteria which are able to produce BC. Furthering our understanding of how to control BC biosynthesis mechanisms using quorum-sensing systems would be of great value [12,13]. Therefore, in this study, we analyzed the quorum-sensing system in *Gluconacetobacter* strains by detecting AHL quorum-sensing signal molecules using traditional biological methods based on bacterial biosensors and identifying them by liquid chromatography-tandem mass spectrometry (LC-MS/MS) [14,15]. Addition of GqqA protein (quorum-quenching protein) could block the biosynthesis of BC, which would indicate that quorum sensing could affect the formation of BC. In addition, two LuxR-type proteins were found in the sequenced genome of *Gluconacetobacter xylinus* CGMCC No. 2955. This may help us uncover the relationship between the quorum-sensing system and bacterial cellulose biosynthesis, which will contribute to improving bacterial cellulose production.

## 2. Results

### 2.1. Detection of AHLs with a Biosensor System

*Gluconacetobacter* strains were streaked out perpendicular to *Agrobacterium tumefaciens* A136 on Luria–Bertani (LB) medium agar containing X-Gal (5-Bromo-4-chloro-3-indolyl β-d-galactopyranoside) and incubated at 30 °C; *Pseudomonas aeruginosa* PAK (*P. aeruginosa* PAK), which can produce AHL molecules, was used as the positive control and bacteria-free water with *Agrobacterium tumefaciens* A136 was used as the negative control. *N*-acyl-homoserine lactone production was detected by the generation of a blue pigment by *A. tumefaciens* A136 [15].

**Figure 1:**
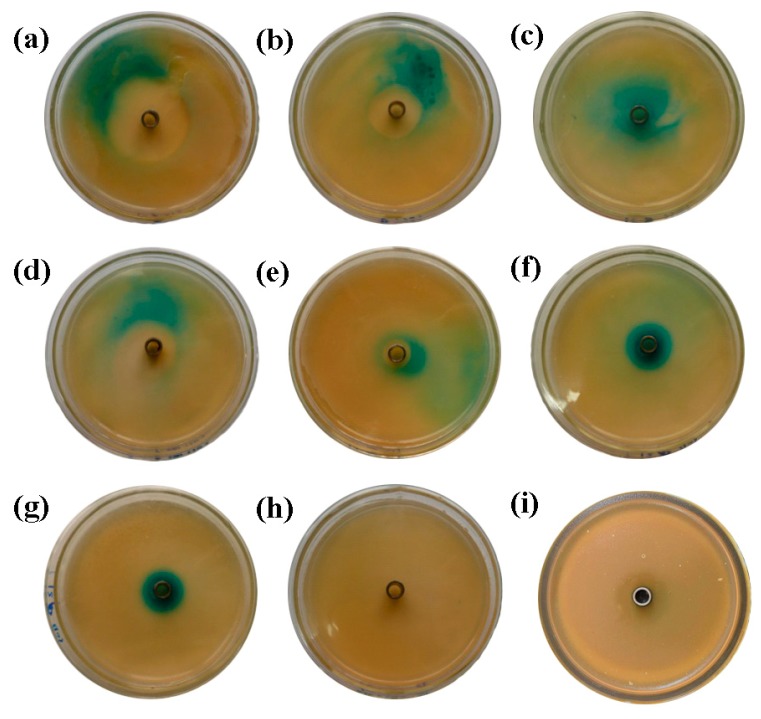
The results of the Oxford cup plate method with bacterial biosensors. (**a**–**c**) Results using extracts from the *Gluconacetobacter sp.* strain SX-1 with the biosensor. (**a**) Undiluted *N*-acyl-homoserine lactone (AHL) extract, (**b**) a 10 fold serial dilution of the AHL extract, and (**c**) a 100 fold serial dilution of the AHL extract. (**d**–**f**) Results using extracts from *G. xylinus* CGMCC no. 2955 that paralleled *Gluconacetobacter sp.* strain SX-1, not repeated here. (**g**) Results using the extracts from *Pseudomonas aeruginosa* PAK (*P. aeruginosa* PAK) with the biosensor, which served as the positive control. (**h**) Bacteria-free water with the biosensor, which served as the negative control. (**i**) Ethanol with the biosensor. (**g**) Results of the Oxford cup method using a 100 fold serial dilution of the AHL extract of *P. aeruginosa* PAK, which served as the positive control. (**i**) Results of the Oxford cup method using pure ethanol, which served as a control to demonstrate that ethanol can produce zones of bacterial inhibition. There were no significant differences between the *Gluconacetobacter* strains and *P. aeruginosa* PAK according to the results of the Oxford cup plate method. This may be caused by a reduction in AHL extract concentration during the preparation process, especially during the extraction and evaporation steps.

The results of the Oxford cup plate method clearly revealed that the *Gluconacetobacter sp*. strain SX-1, *Gluconacetobacter xylinus (G. xylinus)* CGMCC no. 2955, and *P. aeruginosa* PAK all produced AHLs, which served as autoinducers of the quorum-sensing systems in Gram-negative bacteria. A comparison of Figure 1a–c,i clearly demonstrated zones of bacterial inhibition in Figure 1a,b,i, using the Oxford cup method. As the AHL extract was solubilized in ethanol and the biosensor’s tolerance of ethanol was not high, ethanol concentration-dependent zones of bacterial inhibition were apparent; the higher the ethanol concentration, the greater the zone of inhibition. As shown in Figure 1c, when the ethanol was diluted in multiples, the ethanol concentration was low, and thus no zone of inhibition formed. Moreover, the apparent color in these three figures gradually decreased because of the dilution effect. Figure 1d–f presents a similar experimental setup using *G. xylinus* CGMCC no. 2955 instead of *Gluconacetobacter sp.* strain SX-1 and the result was similar to Figure 1a–c.

### 2.2. Identification of AHLs with LC-MS/MS

**Figure 2:**
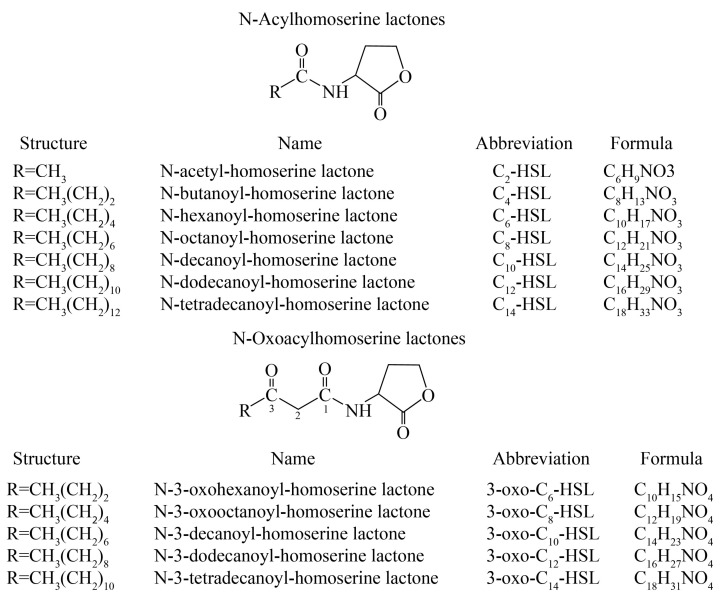
Structures, names, abbreviations, and formulas of the 12 quorum-sensing system AHLs.

Instrumental analysis has incomparable advantages compared with the biosensor system method. Gram-negative bacterial quorum-sensing signal molecules can be analyzed using the bacterial biosensor system method, as well as by modern instrumental analysis such as high-performance liquid chromatography-mass spectrometry (HPLC-MS) [16]. The molecular structures of the studied AHLs differed in the length of the acyl side-chain and the presence or absence of an oxo function on the third carbon (Figure 2). This leads to variations in molecular polarity, allowing the separation of HSLs by reversed-phase HPLC [17].

All AHLs of the Gram-negative bacterial quorum-sensing systems contained a homoserine lactone ring and acyl side chains consisting of 2 to 14 carbons (Figure 2). The lactone ring structure characteristics were not destroyed during mass spectrometry analysis; they remained in one of two characteristic fragments at *m*/*z* 102. Another typical structure feature of AHLs is their relative molecular mass of 172 + 14 n [18]. Based on this feature, the AHLs were directly detected and their structure was confirmed using HPLC-MS and HPLC-MS/MS with 3-oxo-C12-HSL (*m*/*z* 298.2) as the standard sample.

**Figure 3:**
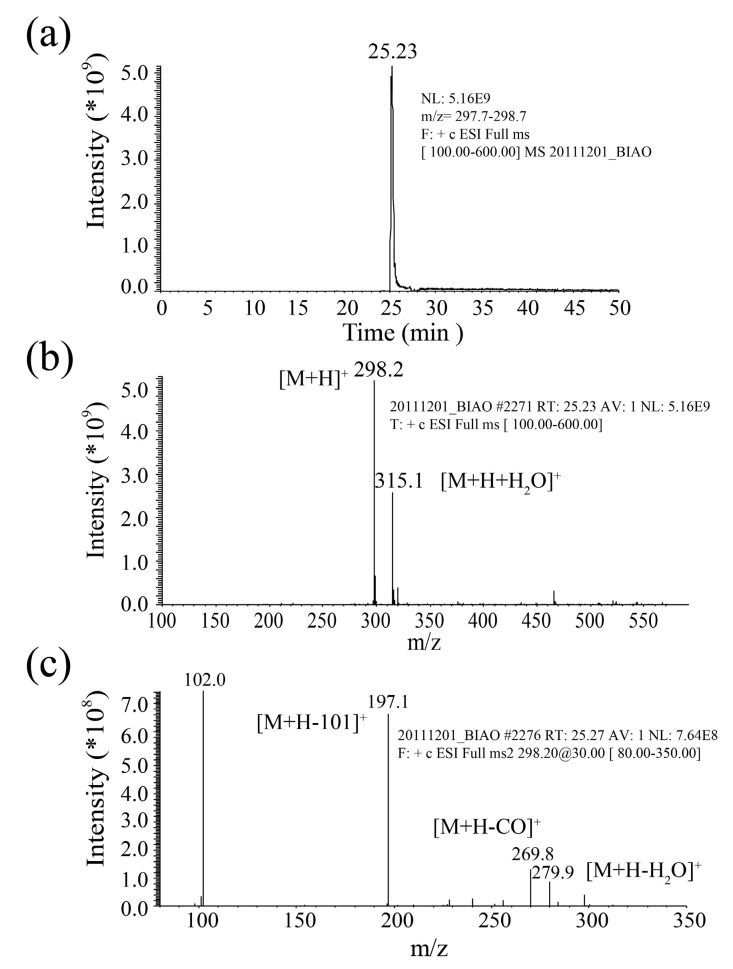
HPLC-MS/MS chromatograms of 3-oxo-C12-HSL standard. (**a**) HPLC chromatograms. (**b**) MS spectrum. (**c**) MS-MS spectrum.

Figure 3 shows the LC-MS/MS results for the 3-oxo-C12-HSL standard (*m*/*z* 298) used to determine the AHL retention time range of the signal molecules. Fragmentation of the parental ion at *m*/*z* 298.2 by LC-MS/MS generated product ions at *m*/*z* 102.0, representing a homoserine lactone moiety; at *m*/*z* 197.1, representing a dodecane acyl side chain replaced by one oxygen; and at *m*/*z* 315.1, representing an (M + H + H_2_O)^+^ ion. In addition, the results of the two spectra standards in the *m*/*z* 269.8 and *m*/*z* 279.9 at two characteristic fragments, representing the (M + H − CO)^+^ ions and (M + H − H_2_O)^+^ ion.

By combining the results of the current paper with a previous study examining the retention time of characteristic AHL fragments [19], we were able to detect the AHLs in *Gluconacetobacter sp.* strain SX-1, *G. xylinus* CGMCC no. 2955, as well as in the positive control *P. aeruginosa* PAK by LC-MS/MS (Table 1).

Analysis of the results in Table 1 demonstrated that there were seven types of AHL autoinducers in the *Gluconacetobacter sp*. strain SX-1. Using the same method, six types of AHL signaling molecules were detected in the fermentation extract from *G. xylinus* CGMCC no. 2955 and *P. aeruginosa* PAK. The MS-MS spectra of the AHLs extracted from the three strains were shown in Supplementary Materials Figures S1–3. Through analysis of the spectra and the resulting peaks, the relative abundance of 3-oxo-C10-HSL in *G. xylinus* CGMCC no. 2955 and C10-HSL in *Gluconacetobacter sp*. strain SX-1 was relatively higher than the other HSLs.

The experiment detected a new type of AHL called C2-HSL that had never been reported before, and it is very noteworthy that a fragment ion of 102 was found in the mass spectrometry analysis. Whether this substance constitutes an AHL remains to be elucidated in future studies.

### 2.3. Impact of GqqA Protein on the Biosynthesis of Cellulose in the Strain *G. Xylinus* CGMCC no. 2955

**Figure 4:**
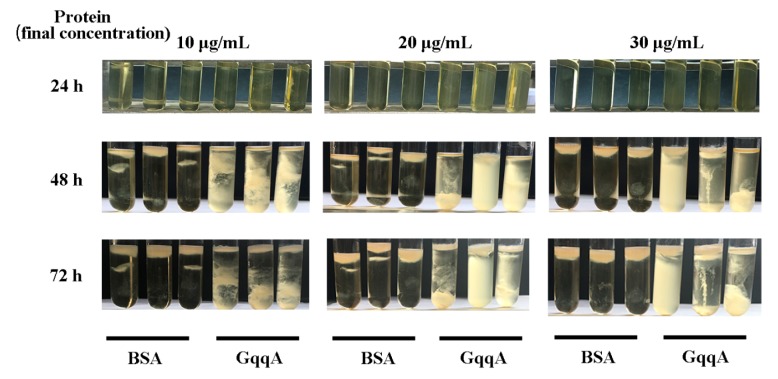
Bacterial cellulose (BC) production phenotypes of the strain *G. xylinus* CGMCC no. 2955 treated with three different concentrations (10, 20, and 30 μg/mL) of the quorum-quenching protein (GqqA protein) and BSA as a control after 24, 48 and 72 h.

Bacterial cellulose is a polysaccharide produced by several microorganisms, particularly *G. xylinus.* Cellulose is always formed in the air liquid interface. In order to verify if AHLs influence the formation of BC, the quorum-quenching protein GqqA, which was reported to be involved in bacterial quorum quenching and cellulose formation [20], was added at three different concentrations to the growth medium of *G. xylinus* CGMCC no. 2955. The cellulose produced by GqqA-treated cells was obviously different from the BSA controls (Figure 4). The culture became turbid in the presence of GqqA, which indicated that the GqqA-altered cellulose aggregated during the culture of *G. xylinus* CGMCC no. 2955. Exogenous signals and quorum-quenching activity led to a depression of BC synthesis.

## 3. Discussion

Our results matched well with previous Japanese studies regarding the relationship between *Gluconacetobacter* strains and quorum-sensing systems [21,22,23]. These studies identified a GinI/GinR quorum-sensing system in *Gluconacetobacter intermedius* NCI1051, similar to the LuxI/LuxR system in *Vibrio fischeri*, which represses oxidative fermentation including acetic acid and gluconic acid fermentation, as well as antifoam activity [21]. An 89 amino acids protein, GinA, the production of which is induced by the quorum-sensing system, regulates the expression of genes such as *pdeA* and *pdeB* [23]. The genes of *pdeA* (encoding a putative cyclic-di-GMP phosphodiesterase) and *pdeB* (encoding a putative phosphodiesterase/diguanylate cyclase) play important roles in the cyclic-di-GMP biosynthetic pathway [24,25,26]. Cyclic-di-GMP is involved in the reversible activation of cellulose synthase, which is the key enzyme in the direct pathway of BC biosynthesis. Taken together, these findings suggest the existence of a relationship between BC biosynthesis and quorum-sensing systems in *Gluconacetobacter* strains.

Seven types of AHL signaling molecules were detected in *Gluconacetobacter sp.* strain SX-1 and six types of AHL signaling molecules were detected in the fermentation extract of *G. xylinus* CGMCC no. 2955 and *P. aeruginosa* PAK. Due to the lower abundance of AHLs in the culture conditions used in this study or technical limitations, there may be other AHLs which exist in the culture that were not detected. Through the NCBI Prokaryotic Genome Annotation Pipeline (released 2013), we obtained the *luxR* gene sequence (NCBI protein_id: ATU74371.1). The *luxR* gene sequence was aligned using BioEdit software version 7.0.9 and searched for sequence similarity. Another LuxR-type protein AsaR (NCBI protein_id: ATU72277.1) was found in the genome of *G. xylinus* CGMCC no. 2955, which showed 28% identities and 43% positives. Multiple-amino-acid alignment of these two LuxR-type proteins against *V. fischeri, P. aeruginosa,* and other *Gluconacetobacter* strains using Clustalx software is shown in Supplementary Materials Figure S4. The LuxR protein (NCBI protein_id: ATU74371.1) is reported as having nine invariant amino acid residues (W57, Y61, D70, P71, W85, G113, E178, L182, and G188) [11], but the AsaR protein only has six of the nine conserved amino acids (W57, Y61, D70, P71, L182, and G188). In addition, due to the low homology of AHL synthetase with the other *luxI* genes, no *luxI* genes in the vicinity of *luxR*/*asaR* were found in the chromosome. Thus, the LuxR and AsaR proteins in *G. xylinus* CGMCC no. 2955 may belong to LuxR solos [11]. Further study will focus on the identification of AHL synthetases and construction of AHL synthetase mutant strains to research which AHLs play important roles in BC biosynthesis and how BC formation is regulated by the AHL-mediated QS system. This will help us discover the relationship between the quorum-sensing system and bacterial cellulose biosynthesis, which will contribute to improving the bacterial cellulose production.

## 4. Materials and Methods

### 4.1. Bacterial Strains and Culture Conditions

*Gluconacetobacter sp.* SX-1 was isolated from traditional Chinese fermented food—Shanxi mature vinegar—and stored at the Key Laboratory of Industrial Fermentation Microbiology, Tianjin University of Science and Technology [27]. *Gluconacetobacter xylinus (G. xylinus)* CGMCC No. 2955 was isolated by our group and stored at the China General Microbiological Culture Collection Center. *Agrobacterium tumefaciens* A136 (with plasmids pCF218 and pCF372), a bioassay strain for a range of AHLs [14,28], was provided by Robert J.C. McLean. *Pseudomonas aeruginosa* PAK which can synthesize AHL molecules [29,30], and which was used as the positive control for the AHL bioassay, was a kind gift from Hong-Jiang Yang at the Key Laboratory of Industrial Fermentation Microbiology, Tianjin University of Science and Technology.

The yeast extract-tryptone-glucose (YPG) medium consisted of 7.5 g of yeast extract, 10 g of tryptone, and 25 g of glucose per 1 L of distilled water, with the addition of 10 g of Na_2_HPO_4_. The *Gluconacetobacter* strains were grown at 30 °C in YPG medium with 1% (*vol*/*vol*) acetic acid and with or without 3% (*vol*/*vol*) ethanol.

### 4.2. Biosensor System Method

An agar well-diffusion assay and β-galactosidase activity assay based on a biosensor were used for qualitative analysis of AHLs. This is a traditional and powerful method for investigating quorum-sensing systems in Gram-negative bacteria [31].

In this method, extracts of the *Gluconacetobacter* strains and *P. aeruginosa* PAK were first obtained from 100 mL of logarithmic phase or stationary phase bacterial liquid cultures (OD660~1). Bacteria were removed by centrifugation at 3000 rpm for 15 min at 4 °C. Supernatants were extracted three times with the same volume of ethyl acetate containing 0.5% formic acid and the mixture was then vortexed for three minutes [15,32]. The emulsion phase was removed using the ultrasound method [33]. The ethyl acetate extracts were dried by evaporation in a rotary evaporator (YARONG RE-3000, Shanghai, China). The residues were dissolved in 2 mL of analytical grade ethanol and diluted 10 and 100 fold with sterile distilled water. The extracts were stored at −20 °C until further analysis. An aliquot of the ethyl acetate extract was placed into an Oxford cup and then in the center of an agar plate coating with *Agrobacterium tumefaciens* A136 cells and 5-Bromo-4-chloro-3-indolyl-β-d-galactopyranoside (X-gal, 40 μg/mL); AHL production was detected by the production of a blue pigment [15,32] by the biosensor *A. tumefaciens* A136. The AHL biosensor *A. tumefaciens* A136, carrying TraR-regulated *traI-lacZ* fusion genes, can produce a blue pigment in the presence of X-Gal in response to exogenous AHLs.

### 4.3. LC-MS Method

Extracts for analytical LC-MS were prepared as described in Section 4.2, except that the residues were dissolved in 2 mL of HPLC-grade methanol. The extracts were stored at −20 °C until further analysis. Samples were filtered through a 0.22 μm polytetrafluoroethylene (PTFE) syringe filter prior to use.

The LC-MS was performed using a Thermo Finnigan LCQ Deca XP MAX system (Thermo Finnigan, San Jose, CA, USA), LC pump, and autosampler. Twenty microliters of the methanol dissolved extracts were applied onto an analytical C18 reversed-phase column (Venusil XBP, 150 × 2.1 mm, particle size 5 μm). Smaller injected volumes were tested without improving peak widths. The elution procedure consisted of an isocratic profile of methanol–water (40:60, *vol*/*vol*) for 10 min, followed by a linear gradient from 40 to 90% methanol in water over 15 min, and an isocratic profile over 20 min. The LC flow rate was 0.2 mL/min [17,34].

The LC-separated compounds were detected by electrospray ionization ion trap mass spectrometry (ESI-MS) under positive-ion conditions. For each compound, two ions were formed: the (M + H)^+^ ion and the (M + Na)^+^ ion. The (M + H)^+^ ion was isolated for MS-MS fragmentation. The MS-MS chromatographic analysis was segmented for the isolation and fragmentation of the eluted (M + H)^+^ ion. The electrospray used nitrogen as the desolvation gas and cone gas and argon as the collision gas. The drying temperature was 300 °C and the voltage of the capillary was 4.5 kV [17,34]. Using the ESI-MS mode, detection of all 12 AHLs was possible with one chromatographic injection (analysis time 45 min); one novel AHL (*m*/*z* = 144) was identified in this study. Full-scan mode detection was used with a scan range of *m*/*z* 50 to 600. The software used was Xcalibur (Version 2.06, Thermo Fischer). The AHL standard 3-oxo-C12-HSL was purchased from Sigma.

### 4.4. Effects of Quorum-Quenching Protein GqqA on the Cellulose Production of CGMCC no. 2955

In this study, a quorum-quenching protein GqqA was used to verify the relationship between BC and AHLs. The expression and purification of GqqA protein was according to the reported method with some modification [20]. The GqqA protein-coding gene *gqqA* was synthesis by Genewiz Biotechnology Co., Ltd. (Suzhou, China). Then, the *gqqA* gene was cloned into the expression vector pETDuet-1, yielding pETDuet-1:GqqA. This recombinant vector was transformed into the strain BL21(DE3) of *Escherichia coli (E. coli)*, which was grown at 37 °C in LB medium with ampicillin to an OD600 = 0.5–0.8. Expression of GqqA was induced by the addition of 0.8 mM IPTG (isopropyl-β-d-1-thiogalactopyranoside), and cultures were incubated overnight at 28 °C and 150 rpm. Cells were harvested by centrifugation at 10,000 rpm for 15 min and 4 °C and resuspended in LE Buffer (50 mM Na_2_HPO_4_; 300 mM NaCl). Disruption of cells was performed using an ultrasonic homogenizer (Scientz-IID, Ningbo Scientz Biotechnology Co., Ningbo, China) at kHz. The disruption period was 3 s with 6 s intervals in an ice bath for a duration of 40 min. The lysate was centrifuged at 11,000 rpm for 20 min and 4 °C. The supernatant obtained was purified using High Affinity Ni-NTA Resin (GenScript, Nanjing, China) following the manufacturer’s protocol. The purified protein was determined by sodium dodecyl sulfate-polyacrylamide gel electrophoresis (SDS-PAGE) and the concentration using the Total Protein Quantitative Assay Kit (Nanjing Jiancheng Biotech., Ltd., Nanjing, China).

For analysis of the effects of the GqqA protein on BC production, *G. xylinus* CGMCC no. 2955 was inoculated in tubes for a final OD600 = 0.02 with 5 mL of YPG medium. The purified GqqA protein extract was added to the tubes with a final concentration of 10 μg/mL, 20 μg/mL, and 30 μg/mL, respectively. Controls were performed with BSA. All experiments were carried out in triplicate.

## 5. Conclusions

Using traditional biological methods based on bacterial biosensors and modern instrumental analysis (liquid chromatography-tandem mass spectrometry), we confirmed the existence of *N*-acyl -homoserine lactones, which are quorum-sensing system autoinducer signaling molecules, in the fermentation fluid of *Gluconacetobacter sp.* strain SX-1. Seven types of AHL signaling molecules were detected in the *Gluconacetobacter sp.* strain SX-1 and six types of AHL signaling molecules were detected in the fermentation extract of *G. xylinus* CGMCC no. 2955. These experimental results prove the existence of a quorum-sensing system in *Gluconacetobacter* strains. Based on the types of AHL signaling molecules, we concluded that the system is similar to the LuxI/LuxR-type quorum-sensing system in *Vibrio fischeri*.

Therefore, in order to regulate the biosynthesis of BC systematically, it is necessary to investigate the correlation between the quorum-sensing system and BC biosynthesis in typical *Gluconacetobacter* strains. These findings would have extensive application potential for improving BC production. Future studies will be needed to elucidate the important issue of controlling BC biosynthesis via quorum-sensing systems.

## Figures and Tables

**Table 1:** Quorum-sensing system AHL signaling molecules *.

No.	Abbreviation	Precursor Ion (*m*/*z*)	Peak Intensity	Retention Time (min)
1	C2-HSL	144	1.04 × 10^4^	1.91
2	C4-HSL	172	3.90 × 10^4^	2.54
3	C6-HSL	200	4.78 × 10^3^	8.30
4	3-oxo-C8-HSL	242	2.07 × 10^3^	12.95
5	C10-HSL	256	2.08 × 10^6^	19.46
6	C12-HSL	284	3.03 × 10^4^	23.81
7	C14-HSL	312	1.60 × 10^4^	29.54
8	C2-HSL	144	4.74 × 10^3^	2.11
9	C4-HSL	172	5.80 × 10^4^	2.68
10	C6-HSL	200	1.53 × 10^3^	8.19
11	3-oxo-C10-HSL	270	8.04 × 10^5^	17.23
12	C12-HSL	284	4.79 × 10^4^	23.77
13	C14-HSL	312	6.70 × 10^3^	29.46
14	C2-HSL	144	5.49 × 10^6^	1.90
15	C4-HSL	172	8.07 × 10^7^	2.56
16	C6-HSL	200	1.32 × 10^6^	8.00
17	C10-HSL	256	4.18 × 10^6^	22.33
18	C12-HSL	284	1.68 × 10^6^	24.59
19	3-oxo-C12-HSL	298	8.92 × 10^4^	26.18

* AHLs of the quorum-sensing system in *Gluconacetobacter sp.* strain SX-1 (No. 1-7), *Gluconacetobacter xylinus* CGMCC No. 2955 (No. 8-13), and *P. aeruginosa* PAK (No. 14-19).

## Supplementary Materials

The following are available online.
