# Supplementary material for: Identification of Quorum-Sensing Molecules of N-Acyl-Homoserine Lactone in Gluconacetobacter Strains by Liquid Chromatography-Tandem Mass Spectrometry
Source: Molecules. 2019 Jul 24;24(15):2694. doi: 10.3390/molecules24152694 (PMC6695853; doi:10.3390/molecules24152694)
Supplement: Supplementary file 1 [file molecules-24-02694-s001.pdf]

molecules

Identification of Quorum Sensing Molecules of N-acyl-Homoserine Lactone in *Gluconacetobacter* Strains by Liquid Chromatography-Tandem Mass Spectrometry

Ling-Pu Liu[1,2], Long-Hui Huang[1,2], Xiao-Tong Ding[1,2], Lin Yan[1,2], Shi-Ru Jia[1,2], Yu-Jie Dai[1,2],Yan-Yan Xie[1,2], Cheng Zhong[1,2,*]

[1] State Key Laboratory of Food Nutrition & Safety, Tianjin University of Science & Technology, Tianjin, P.R. China
[2] Key Laboratory of Industrial Fermentation Microbiology, (Ministry of Education), Tianjin University of Science & Technology, Tianjin, P.R. China
* Correspondence: E-mail: czhong@tust.edu.cn; chzhong.tju@gmail.com; Tel.: +86-22-60601268; Fax: 86-22-60602298

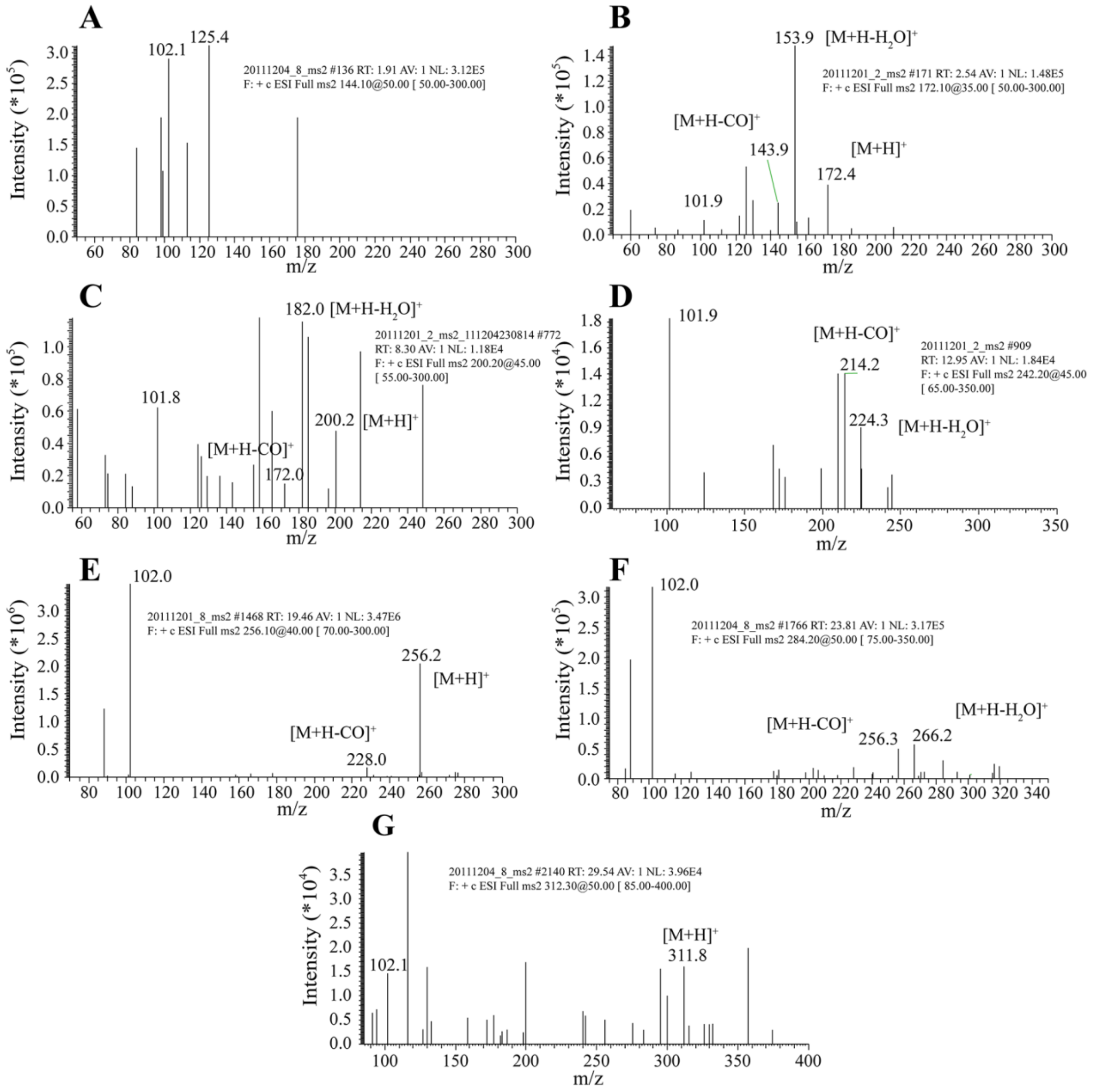

Figure S1: MS-MS fragmentation of AHLs extracted from supernatants of *Gluconacetobacter sp.* strain SX-1. (A) C2-HSL; (B) C4-HSL; (C) C6-HSL; (D) 3-oxo-C8-HSL; (E) C10-HSL; (F) C12-HSL; (G) C14-HSL

molecules

MDPI

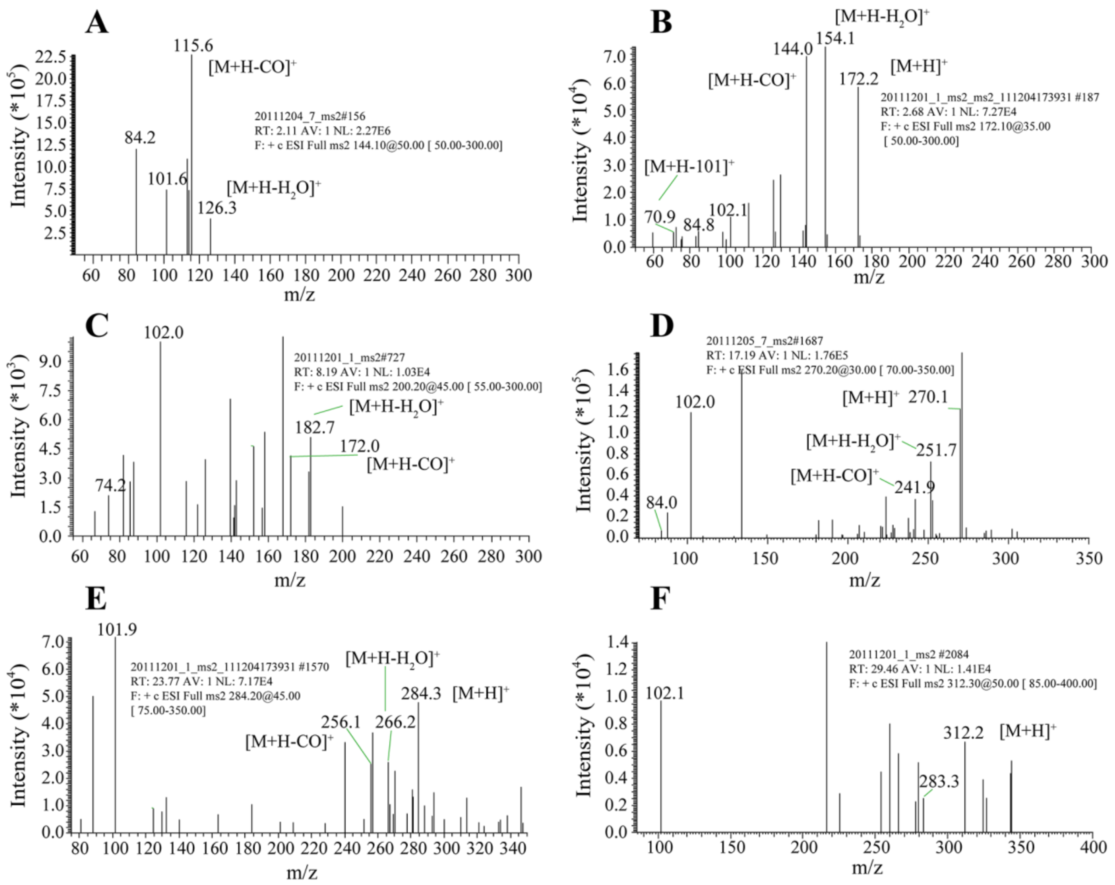

Figure S2. MS-MS fragmentation of AHLs extracted from supernatants of *G. xylinus* CGMCC no. 2955. (A) C2-HSL; (B) C4-HSL; (C) C6-HSL; (D) 3-oxo-C10-HSL; (E) C12-HSL; (F) C14-HSL.

molecules

MDPI

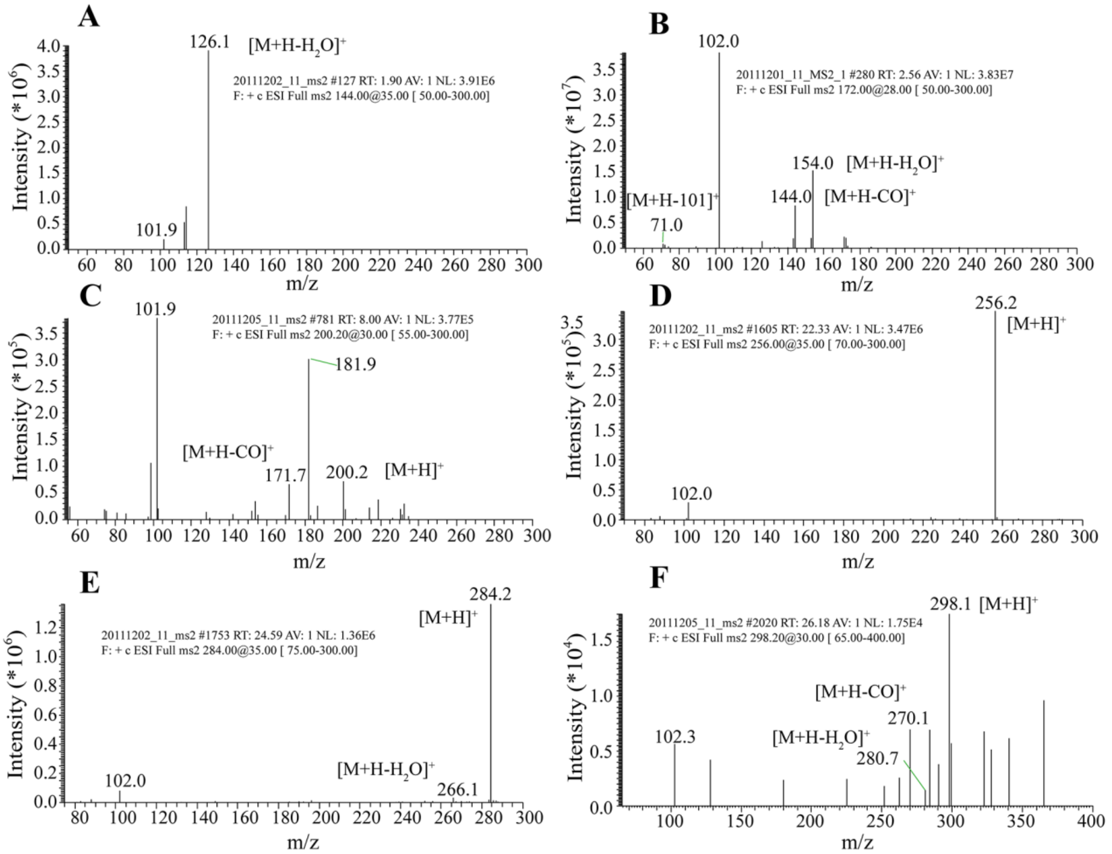

Figure S3: MS-MS fragmentation of AHLs extracted from supernatants of *Pseudomonas aeruginosa PAK*. (A) C2-HSL; (B) C4-HSL; (C) C6-HSL; (D) C10-HSL; (E) C12-HSL; (F) 3-oxo-C12-HSL.

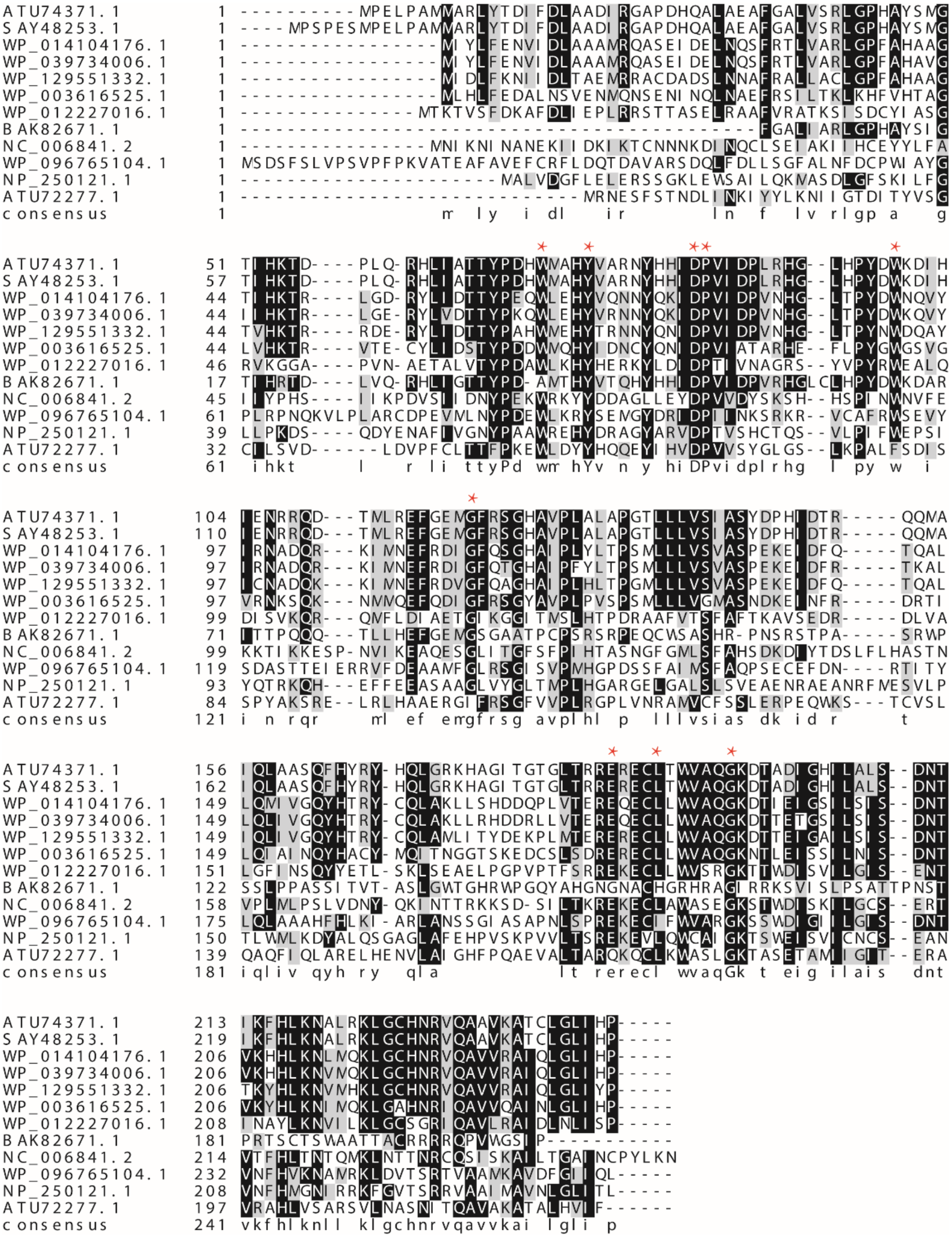

Figure S4. .Multiple-amino-acid alignment of LuxR proteins from *G. xylinus* CGMCC no. 2955 and other bacterias. (ATU74371.1: *G. xylinus* CGMCC no. 2955; SAY48253.1: *Komagataeibacter rhaeticus* iGEM; WP_014104176.1: *Komagataeibacter medellinensis*; WP_039734006.1: *Komagataeibacter intermedius*; WP_129551332.1: *Komagataeibacter xylinus*; WP_003616525.1: *Komagataeibacter hansenii*; WP_012227016.1: *Gluconacetobacter diazotrophicus*; BAK82671.1: *Komagataeibacter medellinensis* NBRC 3288; NC_006841.2: *Vibrio fischeri* ES114; WP_096765104.1: *Rhizobium sophoriradicis*; NP_250121.1: *Pseudomonas aeruginosa* PAO1; ATU72277.1: *Gluconacetobacter xylinus* CGMCC no. 2955) Red asterisks indicate the nine conserved amino acids.
